# Palmatine Ameliorates Motor Deficits and Dopaminergic Neuron Loss by Regulating NLRP3 Inflammasome through Mitophagy in Parkinson's Disease Model Mice

**DOI:** 10.1007/s12035-024-04367-2

**Published:** 2024-08-03

**Authors:** Jindong Zhao, Ji Wang, Kunying Zhao, Shuda Yang, Junfang Dong, Yuxiao Zhang, Shangpeng Wu, Lirong Xiang, Weiyan Hu

**Affiliations:** 1 School of Pharmaceutical Science & Yunnan Key Laboratory of Pharmacology for Natural Products, Kunming Medical University, Kunming, 650500 PR China; 2 College of Modern Biomedical Industry, Kunming Medical University, Kunming, 650500 PR China; 3 School of Chinese Materia Medica &Yunnan Key Laboratory of Southern Medicine Utilization, Yunnan University of Chinese Medicine, Kunming, 650500 PR China

**Keywords:** Palmatine, Mitophagy, NLRP3 inflammasome, Parkinson’s disease

## Abstract

NLRP3 inflammasomes-mediated proinflammatory response and mitochondrial dysfunction play a critical role in the etiology and pathogenesis of Parkinson's disease. Negative regulation of NLRP3 inflammasome activation through mitophagy may be an important strategy to control NLRP3 inflammasome-mediated proinflammatory responses. Palmatine (PAL), an isoquinoline alkaloid found in various of plants, has potent pharmacological effects such as anti-inflammatory and anti-oxidation. However, the specific role of PAL in the pathology of Parkinson's disease remains unclear. In this study, we found that treatment with PAL improved motor deficits and reduced the loss of dopaminergic neurons in MPTP mice. Further results showed that PAL promoted mitophagy and inhibited the proinflammatory response mediated by NLRP3 inflammasomes. In addition, chloroquine (CQ, mitophagy inhibitor) attenuated the ameliorative effects of PAL on the motor deficits and dopaminergic neuron damage, as well as the inhibitory effect of PAL on NLRP3 inflammasome. Collectively, these results provide strong evidence that PAL ameliorates motor deficits and dopaminergic neuron death in Parkinson’s disease, and the mechanism may be related to its inhibition of NLRP3 inflammasome activation via promoting mitophagy.

## Introduction

Parkinson's disease (PD) is a common chronic progressive neurodegenerative disease with a prevalence of about 2–3% in people over 65 years old [1–3]. The clinical manifestation of PD is a motor syndrome characterized by bradykinesia, tremor balance disorder, resting tremor and muscle rigidity [4, 5]. At the same time, patients with PD may suffer from hyposmia, sleep and cognitive impairment, neuropsychiatric features, and dementia [6, 7]. It is characterized by the loss of nigrostriatal dopaminergic neurons, misfolded aggregation of α-synuclein and formation of Lewy bodies [8]. Though the exact etiology and pathogenesis of PD are not fully understood, strong evidence indicates that mitochondrial dysfunction [9], oxidative stress [10], and neuroinflammation [11] significantly contribute to the PD's pathological progression [12].

Neuroinflammation is an immune response activated by microglia and astrocytes in response to infection, toxic stimulation or autoimmune signal stimulation [13, 14]. NLRP3 inflammasome is one of the most characterized and widely studied inflammasomes [15]. NLRP3 inflammasome is overactivated in the brain tissue of PD patients, leading to the aggravation of inflammatory response and neuronal damage [16]. Mitophagy is an important quality control mechanism to remove damaged or excess mitochondria [17, 18]. Mutations in various genes related to the pathogenesis of PD(such as α-synuclein [19, 20], PINK1 [21, 22], Parkin [23, 24], DJ-1 [25], LRRK2 [17, 26], ATP13A2 [27], HTRA2) lead to impaired mitophagy and disrupt mitochondrial homeostasis. In the autopsy of PD animal models and PD patients, a large number of swollen and damaged mitochondria were found in nerve cells [28, 29]. These damaged mitochondria cannot be effectively removed and will release excessive ROS and other harmful substances, thereby activating the immune response through the NLRP3 inflammasome [30, 31]. Therefore, inhibiting NLRP3 inflammasome by regulating mitophagy may become an important way to treat PD.

As a natural isoquinoline alkaloid, PAL has pharmacological effects such as antibacterial [32], anti-inflammatory [33], anti-oxidation [34]and regulating immune response [35]. Previous studies have shown that PAL plays a neuroprotective role by reducing inflammatory response, inhibiting oxidative stress [36, 37]. However, the role of PAL against PD have not been explored to date. Therefore, in the present study, we used MPTP-induced PD model mice and MPP^+^/LPS + MPP^+^ stimulated primary neurons or BV2 cells to investigate the effects and possible mechanisms of PAL on behavioral impairment and pathological changes in PD.

## Materials and Methods

### Animals

C57BL/6 male mice (22-25 g) were purchased from the Department of Zoology & Yunnan Key Laboratory of Pharmacology for Natural Products, Kunming Medical University (Kunming, China, SYXK (Yunnan) K2020-006). Animal welfare and experimental procedures were carried out strictly in accordance with the related ethical regulations of Kunming Medical University. All mice were provided with free access to standard laboratory food and water under specific pathogen-free conditions. They were housed at a temperature of 23 ± 2 °C and a relative humidity of 40% to 60%, with a 12-hour light/dark cycle.

### Reagents

PAL (HPLC ≥ 98%) was purchased from HerbSubstance (PCS0470). 1-methyl-4-phenyl-1, 2, 3, 6-tetrahydropyridine hydrochloride (MPTP hydrochloride, M0896), MPP^+^ iodide (D048), levodopa (L-dopa, D9628), dimethyl sulfoxide (DMSO, D8418), CQ diphosphate (C6628), and poly-D-lysine hydrobromide (PDL, P6407) were purchased from Sigma-Aldrich. LPS (bs-8000P) was purchased from BIOS. NO assay kit (S0021M) was purchased from Beyotime. CCK8 cell counting kit (C8022), mouse IL-6 Elia kit (P2816005) and mouse TNF-α Elia kit (P2816004) were purchased from Adamas life. DCFDA/H2DCFDA-Cellular ROS Assay Kit (ab113851), JC-1-Mitochondrial Membrane Potential Assay Kit (ab113850) were purchased from abcam. MitoSOXTM Red mitochondrial superoxide indicator kit (M36008) was purchased from Invitrogen. B27 Supplement (17504044), NeurobasalTM Medium (12348017) and 0.25%Trypsin–EDTA (25200072) were purchased from Gibco. Skim Milk (232100) was purchased from BD-Media.

For western blot analysis and immunofluorescence, we used the following antibodies: anti-PINK1 (D8G3) Ab (1: 1000, Cell Signaling Technology, 6946), anti-LC3A/B Ab (1:1000, Cell Signaling Technology, 4108), anti-NF-κB p65 (D14E12) Ab (1:1000, Cell Signaling Technology, 8242), anti-caspase-1 Ab (1:1000, Cell Signaling Technology, 2225), anti-NLRP3 (D4D8T) Ab (1:1000, Cell Signaling Technology, 15,101), anti-IL-1β Ab (1:1000, Affinity, AF5103), anti-TTMS1/ASC Ab (1:1000, Affinity, AF6304), anti-Iba-1 Ab (1:1000, Abcam, ab178846), anti-Tyrosine Hydroxylase Ab (1:5000, Abcam, ab137869), Goat Anti-Rabbit lgG H& (HRP) (1:5000, Abcam, ab6721), Goat Anti-Mouse lgG H& L (HRP) (1:5000, Abcam, ab6789), Donkey anti-Rabbit IgG (H + L) Highly Cross-Adsorbed Secondary Antibody, Alexa Fluor™ 546 (1:500, Invitrogen, A10040).

### Cells Cultures

The midbrain of the fetuses was removed from the pregnant mice at 18–21 days of gestation under anesthesia. The blood membranes and blood vessels of the midbrain tissue were mechanically stripped off. The cell suspension was obtained by adding 0.25% trypsin for 15 min. Neurons were then seeded uniformly in PDL precoated 6/24/96 well plates, and the medium was changed the next day. Neurons can be used at 8 days.

Cell lines: BV2 cells were purchased from Wuhan Procell Technology Co., LTD. BV2 cells were cultured in 10% fetal bovine serum and 1% penicillin/streptomycin at 37° C in a 5% CO_2_ atmosphere. The medium was changed daily until the cells reached more than 80% density and were subsequently digested and passaged.

Cell model: MPP^+^ is a neurotoxin that selectively affects dopaminergic neurons, causing their degeneration, which is a key feature of PD. By employing MPP^+^ in primary neurons, we aimed to simulate the neuronal damage characteristic of PD. Additionally, the combination of MPP^+^ with LPS in BV2 cells was chosen to replicate the inflammatory conditions often linked to PD, as LPS is known to stimulate microglia, the brain's primary immune cells, and intensify neuronal damage. This approach enables us to investigate both the neurotoxic and inflammatory facets of PD pathology within a controlled environment.

### Cell Viability

Cell viability was detected by CCK8 cell counting kit. In brief [38], cells were seeded in 96-well plates and pretreated with different concentrations of PAL for 2 h followed by MPP^+^/LPS + MPP^+^ for 24 h. Then 10 μl of CCK-8 reagent was added and incubated for 2 h, and finally the absorbance was measured at 450 nm.

### Mitochondrial Membrane Potential

JC-1 is an ideal fluorescent probe widely used to detect mitochondrial membrane potential. At normal membrane potential, JC-1 formed aggregates with high red fluorescence intensity. Damage to the membrane potential, the dye changes from aggregate to monomeric form, red fluorescence decreases and green fluorescence increases. In brief [39], neuronal cells were seeded in 6-well plates and administered at 7 days. The cells were pretreated with 5, 10, 20 μmol/L PAL solution for 2 h, followed by 30 μmol/L MPP^+^ for 24 h. JC-1 working solution sufficient to cover all neuronal cells was added and incubated for 15 min at 37° C in a 5% CO2 incubator. Then the cells were washed twice with 1 × PBS, and 2 ml of medium was added to the cells for observation under fluorescence microscope.

### Mitochondrial ROS

Mitochondrial ROS was detected using the MitoSOX™Red mitochondrial superoxide indicator. Neuronal cells were pretreated with 5, 10, 20 μmol/L PAL solution for 2 h, followed by 30 μmol/L MPP^+^ treatment for 24 h, and incubated with 20 μM MitoSOX™Red for 15 min at 37° C. Cells were washed three times with 1 × HBSS and observed under a fluorescence microscope.

### Animal Treatment

The subacute PD model was established by intraperitoneal injection of MPTP (30 mg/kg) [40]for 7 consecutive days. C57BL/6 mice were treated orally by gavage with PAL (50 mg/kg, 100 mg/kg, 200 mg/kg) [41] or combined with CQ (50 mg/kg, intraperitoneal injection) [42], or Levodopa (120 mg/kg) [43], or 0.5% CMC-Na for 7 consecutive days.

### Behavioral Tests

- (1) Open field test (OFT)
  OFT is used to evaluate the spontaneous activity behavior of mice in novel environments [44]. The open field consisted of a 50 cm × 50 cm × 50 cm plastic box. Mice were placed in the center of the open field and the total distance moved by the mouse during 5 min was recorded. The experiments were conducted in a dark and quiet environment.
- (2) Pole test
  The pole climbing test was used to evaluate the motor coordination ability of mice [45]. A wooden pole of 50 cm long and 1 cm wide was selected for the climbing pole device. The mouse was placed on the pole, and the time it took for the mouse to turn its head down (T-turn) and to descend from the top to the bottom of the pole was recorded.

### Cytokine Analysis by Enzyme-linked Immunosorbent Assay (ELISA)

ELISA kits specific for mouse were used to detect the levels of TNF-α and IL-6 in the serum of different groups. Mouse serum was reacted with ELISA reagent and then incubated with horseradish peroxidase-labeled Streptavidin to form sandwich immune complexes. Signals were detected at 450 nm using a microplate reader, and concentrations were obtained by drawing a standard reference curve.

### Immunofluorescence

After blocking with 10% BSA for 1 h, cells or tissues were incubated with primary antibodies overnight at 4 °C. Then, the tissues or cells were washed three times with PBS at room temperature and incubated with secondary antibodies for 2 h. Finally, they were stained with DAPI and visualized by fluorescence microscopy.

### Immunoblotting

Proteins were separated on polyacrylamide gels (SDS-PAGE) and transferred to polyvinylidene difluoride membranes (PVDF), after which they were blocked with 5% skim milk for 2 h at room temperature. PVDF membranes were incubated with primary antibodies overnight at 4 ℃. Finally, the membranes were incubated in horseradish peroxidase (HRP) conjugated secondary antibody for 2 h, and chemiluminescence imaging was performed using a chemiluminescence imaging system.

### Statistical Analysis

The statistical significance of the differences between groups was determined using unpaired t- test, and one-way analysis of variance (ANOVA) was used for the experiments with multiple groups. The data results were presented as mean ± standard error of the mean (Mean ± SEM). A P-value of < 0.05 is considered statistically significant.

## Results

### PAL Protects Against MPP^+^ -Induced Cytotoxicity and Inhibits NLRP3 Inflammasome Activation

**Fig. 1:**
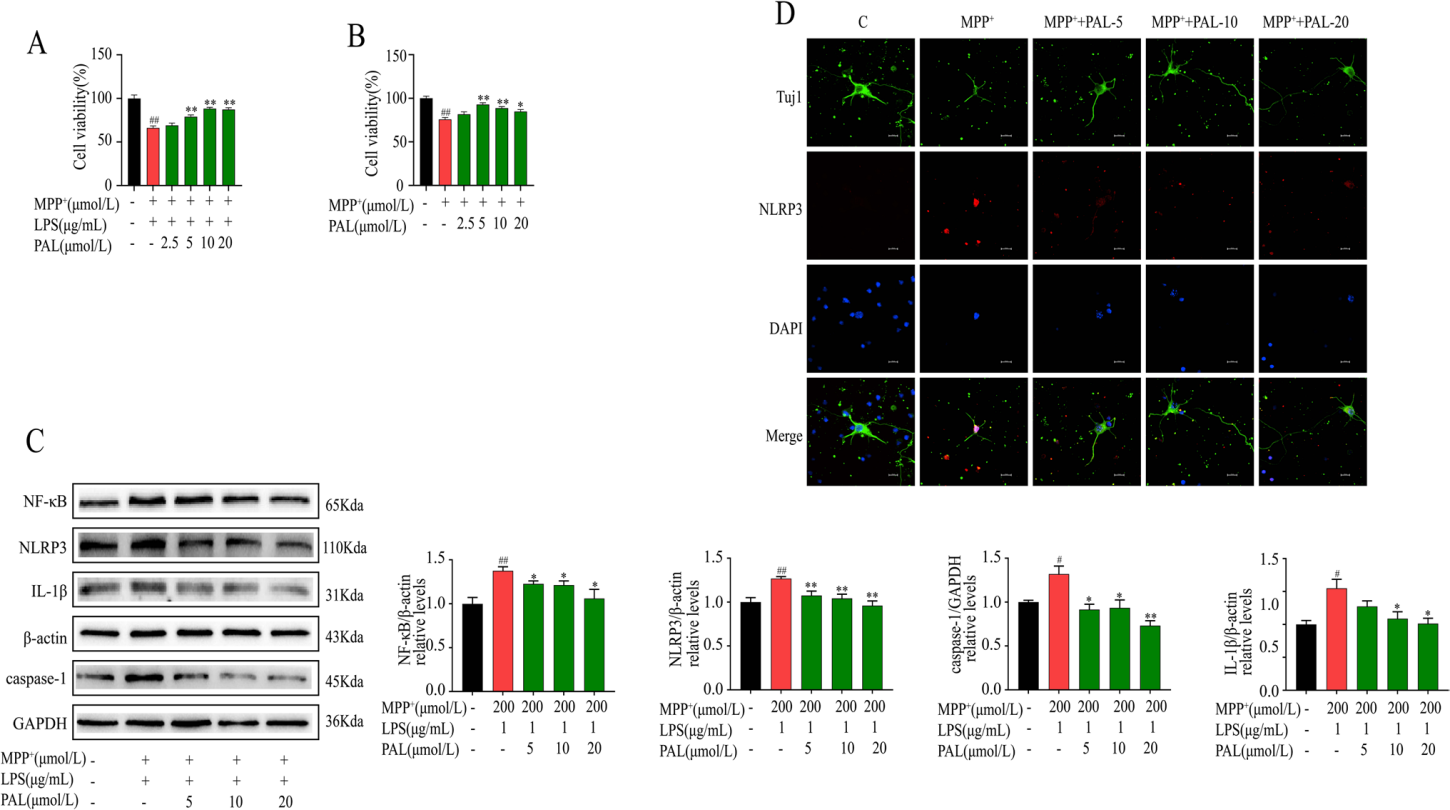
Effects of PAL on MPP^+^/LPS + MPP^+^ -induced survival and NLRP3 inflammasome expression of primary neurons and BV2 cells. (**A**) BV2 cells were pretreated with PAL for 2 h, followed by LPS + MPP^+^ treatment for 24 h, and the cell viability was measured by CCK8. (**B**) Primary neurons were pretreated with PAL for 2 h, followed by MPP^+^ treatment for 24 h, and the cell viability was measured by CCK8. (**C**) western blot was used to detect the effect of PAL on the expression of NF-κB, NLRP3, caspase-1 and IL-1β induced by LPS + MPP^+^ in BV2 cells. (**D**) The expression of NLRP3 was observed by fluorescence microscope. Scale bar, 12.5 μm. Quantified data are normalized to the control group (the control group value is equal to 1). Data are expressed as means ± SEM, (n = 3). ^#^ P < 0.05, ^##^ P < 0.01 vs the saline group. ^*^ P < 0.05, ^**^ P < 0.01 vs MPP^+^ group

To explore the potential protective effect of PAL under neurotoxic conditions, primary neurons and BV2 cells were treated with MPP^+^ or LPS + MPP^+^ for 24 h, and cell viability was measured by CCK-8. We found that PAL significantly enhanced MPP^+^ or LPS + MPP^+^ -induced viability of neurons and BV2 cells (Fig. 1A and B). To evaluate the anti-inflammatory effect of PAL, the expression levels of NF-κB, NLRP3, caspase-1, and IL-1β in LPS + MPP^+^ treated BV2 cells were detected by WB. As shown in Fig. 1C, LPS + MPP^+^ significantly activated the NLRP3 inflammasome and increased the levels of NF-κB, NLRP3, caspase-1, and IL-1β, while the expression of NLRP3 and its associated proteins was inhibited after PAL treatment. Consistently, PAL reduced the fluorescence intensity of NLRP3 in MPP^+^ -treated primary neurons under fluorescence microscopy (Fig. 1D). These results indicate that PAL protects against MPP^+^-induced cytotoxicity and inhibits NLRP3 inflammasome activation.

### PAL Increases the Expression of Mitophagy-related Proteins and Improves Mitochondrial Dysfunction

**Fig. 2:**
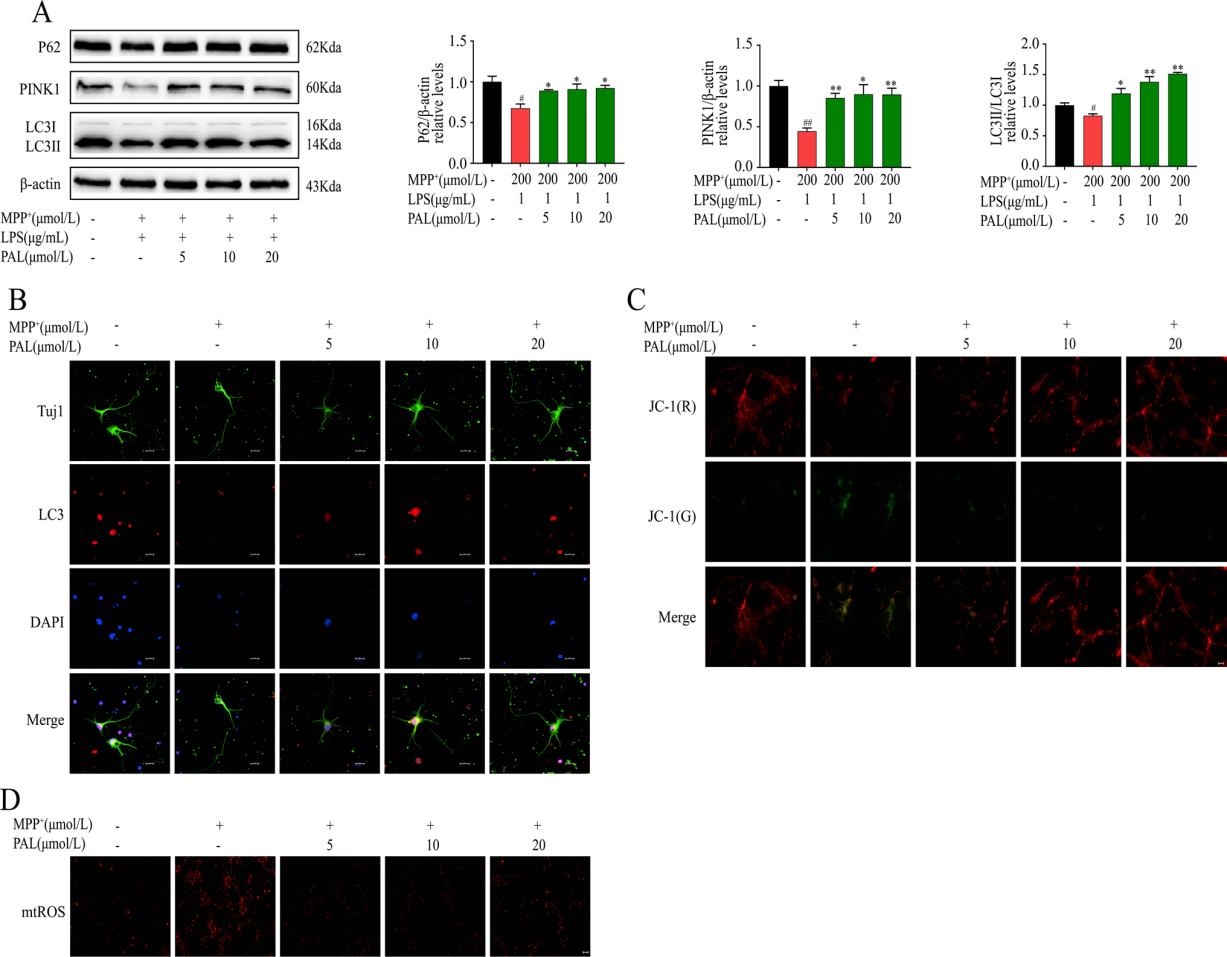
Effect of PAL on mitophagy-related proteins and mitochondrial dysfunction induced by MPP^+^ or LPS + MPP^+^. (**A**)western blot was used to detect the effect of PAL on the expression of LC3, PINK1, P62 induced by LPS + MPP^+^ in BV2 cells. (**B**)The expression of LC3 was observed by fluorescence microscope. Scale bar, 200 μm. (**C**)Mitochondrial membrane potential levels were measured by JC-1 kit. Scale bar, 200 μm. (**D**) Mitochondrial ROS was detected using the MitoSOX™Red mitochondrial superoxide indicator. Scale bar, 12.5 μm. Quantified data are normalized to the control group (the control group value is equal to 1). Data are expressed as means ± SEM, (n = 3). ^#^ P < 0.05, ^##^ P < 0.01 vs the saline group. ^*^ P < 0.05, ^**^ P < 0.01 vs LPS + MPP^+^ group

As shown in Fig. 2, the levels of microtubule-associated protein light chain 3 (LC3)-II and P62, PINK1 levels were significantly reduced in LPS + MPP^+^ treated BV2 cells, which was significantly reversed by PAL treatment (Fig. 2A). Similarly, PAL enhanced LC3 fluorescence intensity in MPP^+^-treated primary neurons under fluorescence microscopy (Fig. 2B).

The above experimental results show that the PAL administration increased the expression of mitophagy-related proteins, which implied that mitochondrial dysfunction might also be ameliorated by PAL. To test this hypothesis, we measured mitochondrial membrane potential by JC-1, which showed that MPP^+^ induced a decrease in membrane potential and a shift of JC-1 from aggregate to monomeric form, with a decrease in red fluorescence and an increase in green fluorescence (Fig. 2C). MitoSox assays showed that MPP^+^ induced an increase in mitochondrial superoxide anion levels (Fig. 2D). Treatment with PAL restores mitochondrial membrane potential and partially inhibits ROS production, thus playing a crucial role in protecting mitochondrial dysfunction.

### PAL Ameliorates Motor Deficits and Dopaminergic Neuron Damage in MPTP-induced Mice

**Fig. 3:**
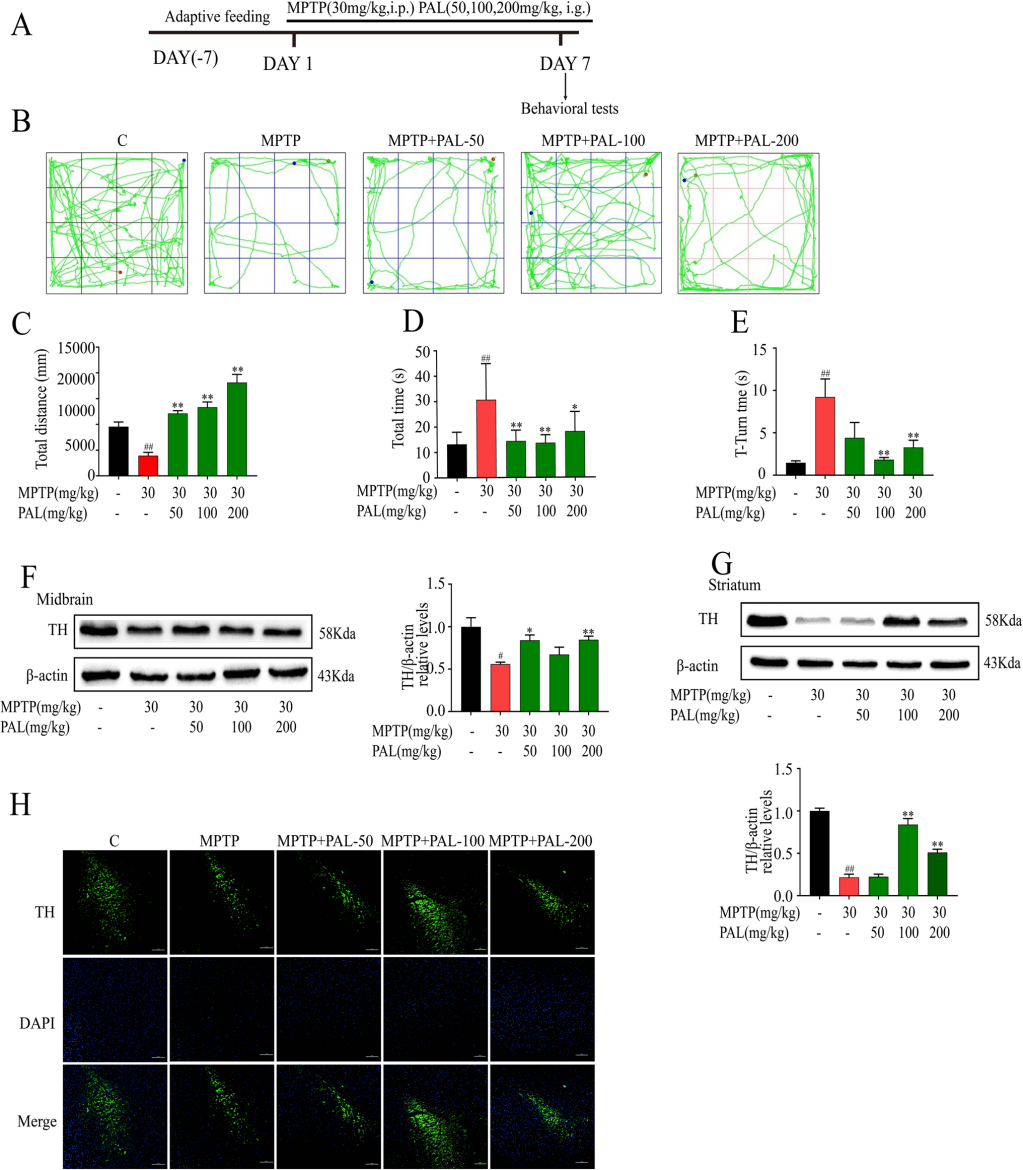
PAL ameliorated motor deficits and protected dopaminergic neurons in MPTP-treated mice. (**A**) The schedule of MPTP-induced mouse PD model. (**B-C**) open field test. (n = 8). (**D-E**) pole test. (n = 8). (**F**) TH expression in the midbrain. (**G**) TH expression in striatum. (**H**) Immunofluorescence detection neurons in striatum. Scale bar, 100 μm. Quantified data are normalized to the control group (the control group value is equal to 1). Data are expressed as means ± SEM, (n = 3). ^#^ P < 0.05, ^##^ P < 0.01 vs the saline group. ^*^ P < 0.05, ^**^ P < 0.01 vs MPTP group

To evaluate the effects of PAL on alleviating motor deficits in PD model mice, behavioral tests were performed. The movement distance of MPTP mice in the open field is significantly reduced, the mice show obvious movement slowness. (Fig. 3B and C). Similarly, on the pole test, MPTP mice spent more time which mice head turned its head down and from the top to the bottom of the pole (Fig. 3D and E). PAL treatment significantly alleviated the motor deficits, restored the motor coordination and improved the bradykinesia of PD mice.

To investigate the effect of PAL on dopaminergic neuron loss, we examined the expression of dopaminergic neurons and TH by immunoblotting and immunofluorescence. As shown in Fig. 3F to H, MPTP-treated mice showed a significant reduction in dopaminergic neurons and decreased expression of TH, while PAL treatment alleviated neuronal loss and increased TH expression.

### PAL Inhibits NLRP3 Inflammasome and Suppresses Microglial Activation

**Fig. 4:**
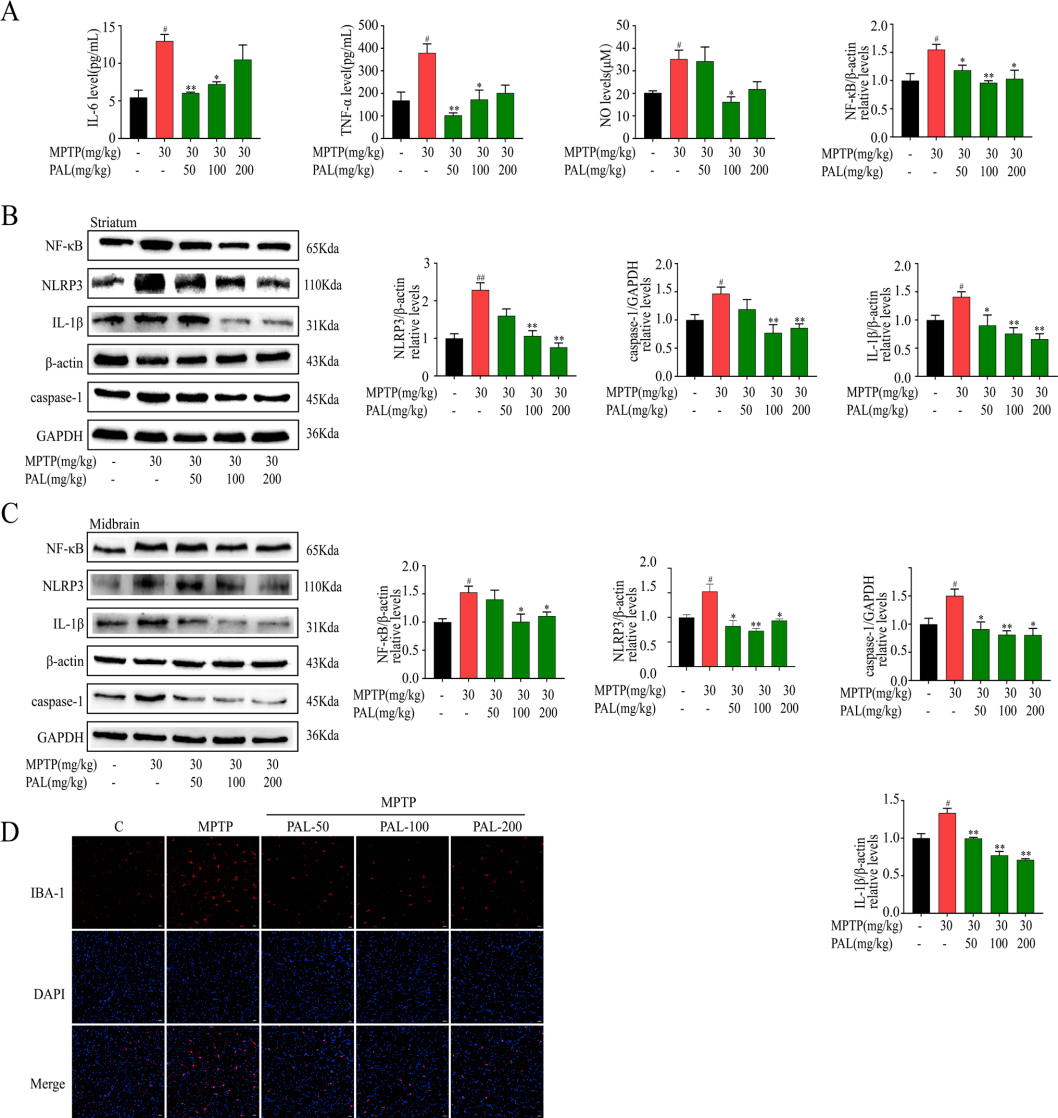
PAL inhibits NLRP3 inflammasome and suppresses microglial activation. (**A**) Expression of proinflammatory cytokines IL-6, TNF-α, and NO. (n = 3). (**B**) western blot was used to detect the expression of NF-κB, NLRP3, caspase-1 and IL-1β in the striatum. (**C**) western blot was used to detect the expression of NF-κB, NLRP3, caspase-1 and IL-1β in the midbrain. (**D**) Immunofluorescence detection of microglia. Scale bar, 20 μm. Quantified data are normalized to the control group (the control group value is equal to 1). Data are expressed as means ± SEM, (n = 3). ^#^ P < 0.05, ^##^ P < 0.01 vs the saline group. ^*^ P < 0.05, ^**^ P < 0.01 vs MPP^+^ group

After confirming the protective effect of PAL on motor deficits and dopaminergic neuron loss in Parkinson's mice, we further examined its anti-inflammatory effect. As shown in Fig. 4, MPTP mice showed increased expression of pro-inflammatory factors IL-6, TNF-α, and NO in the serum of MPTP mice (Fig. 4A), with significant activation of microglia (Fig. 4D). PAL treatment inhibited microglia activation and reduced the expression of pro-inflammatory cytokines IL-6, TNF-α and NO. The immunoblotting results showed that PAL treatment significantly reduced the expression of NF-κB, NLRP3, caspase-1, and IL-1β in the midbrain and striatum of PD model mice (Fig. 4B and C). Taken together, these results suggest that PAL inhibits the activation of NLRP3 inflammasome.

### PAL Improves the Expression of Mitophagy-related Proteins in MPTP-induced Mice

**Fig. 5:**
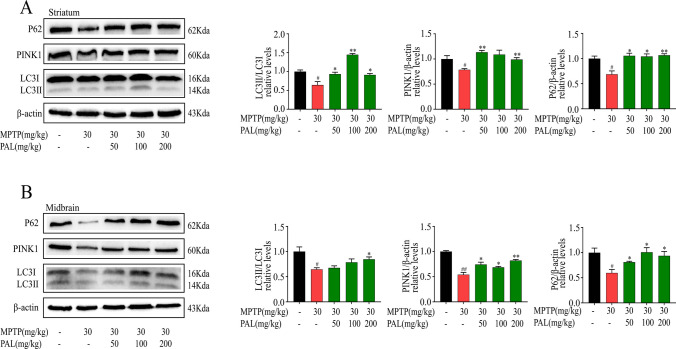
PAL enhanced the expression of mitophagy-related proteins. (**A**) western blot was used to detect the expression of LC3, P62 and PINK1 in the striatum. (**B**) western blot was used to detect the expression of LC3, P62 and PINK1 in the midbrain. Quantified data are normalized to the control group (the control group value is equal to 1). Data are expressed as means ± SEM, (n = 3). ^#^ P < 0.05, ^##^ P < 0.01 vs the saline group. ^*^ P < 0.05, ^**^ P < 0.01 vs MPP^+^ group

Mitophagy is a protective mechanism to remove damaged mitochondria to maintain mitochondrial homeostasis and protect against neuronal damage. We investigated the effect of PAL on the expression of mitophagy-related proteins by immunoblotting in the midbrain and striatum of PD model mice. In the PD model mice, the levels of microtubule-associated protein light chain 3 (LC3)-II, autophagic substrate P62, and mitochondrial marker protein PINK1 were significantly decreased in MPTP mice (Fig. 5A and B). This decrease was reversed by treatment with PAL, which promoted the conversion of LC3 I to LC3 II and increased the expression of P62 and PINK1 proteins.

### CQ Attenuated the Ameliorative Effects of PAL on the Motor Deficits and Dopaminergic Neuron Damage

**Fig. 6:**
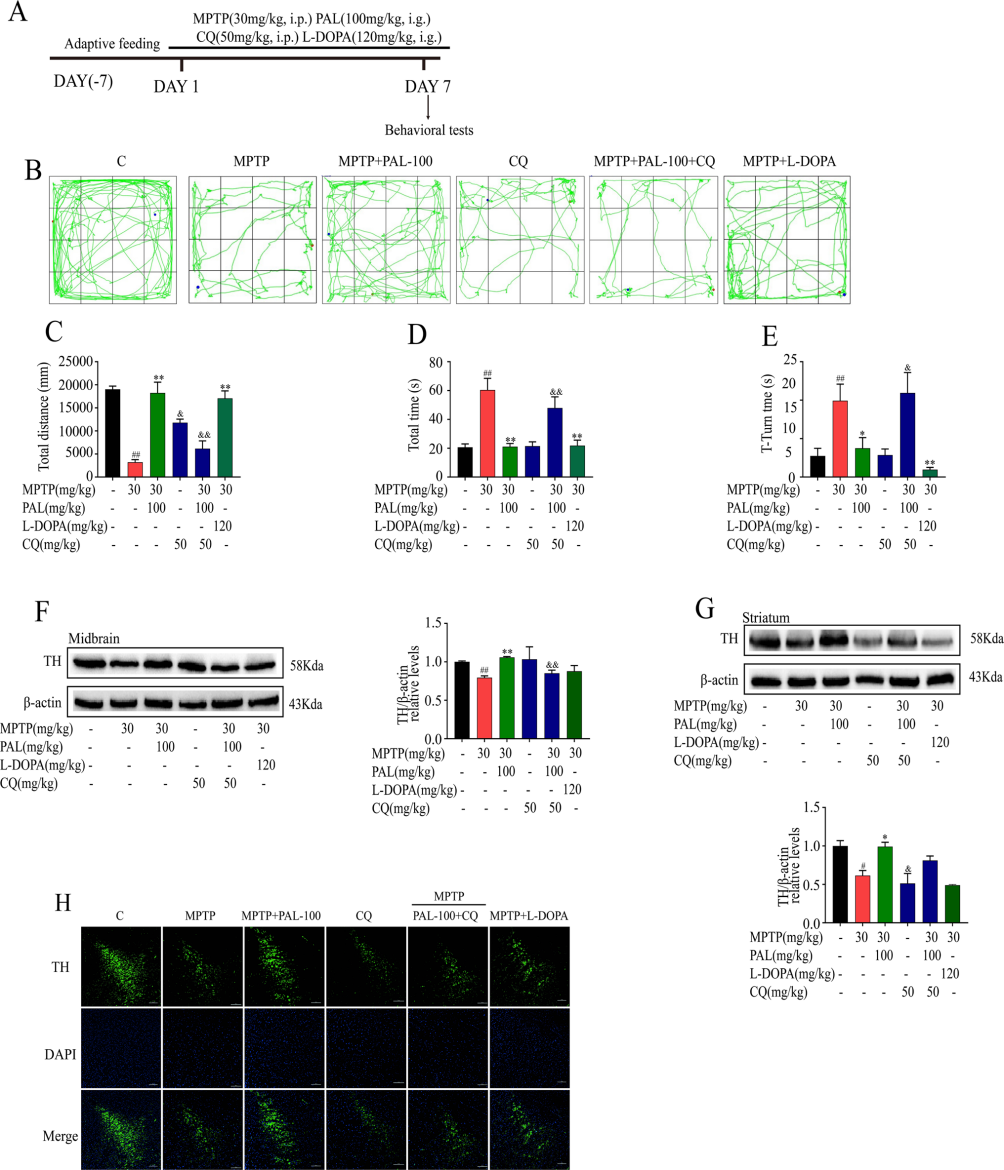
CQ reversed the ameliorative effects of PAL on the motor deficits and dopaminergic reduction in PD mice. (**A**) Timeline of CQ administration. (**B-C**) open field test. (n = 8). (**D-E**) pole test. (n = 8). (**F**) TH expression in the midbrain. (**G**) TH expression in striatum. (**H**) Immunofluorescence detection neurons in striatum. Scale bar, 100 μm. Quantified data are normalized to the control group (the control group value is equal to 1). Data are expressed as means ± SEM, (n = 3). ^#^ P < 0.05, ^##^ P < 0.01 vs the saline group. ^*^ P < 0.05, ^**^ P < 0.01 vs MPTP group, ^&^ P < 0.05, ^&&^ P < 0.01 vs MPTP + PAL-100 group

CQ is a universal mitophagy inhibitor, which inhibits the fusion of mitophagosomes and lysosomes. We used CQ to test the hypothesis that PAL's effects on motor deficits and dopaminergic neuron loss in PD mice are mediated by its influence on mitophagy. As shown in the Fig. 6, CQ abolished the ameliorating effect of PAL on motor deficits in MPTP mice. The distance of movement in the open field was significantly reduced, and the time spent with the head turned downward and from the top to the bottom of the pole were significantly increased (Fig. 6B-E). Meanwhile, CQ abolished the protective effect of PAL on dopaminergic neurons in MPTP mice, decreased TH protein expression, and aggravated dopaminergic neuron damage (Fig. 6F-H). Additionally, L-dopa administration alleviates motor deficits in PD mice but does not significantly improve the damage to dopaminergic neurons (Fig. 6B-H).

### CQ Eliminated the Inhibitory Effect of PAL on NLRP3 Inflammasome and Microglia

**Fig. 7:**
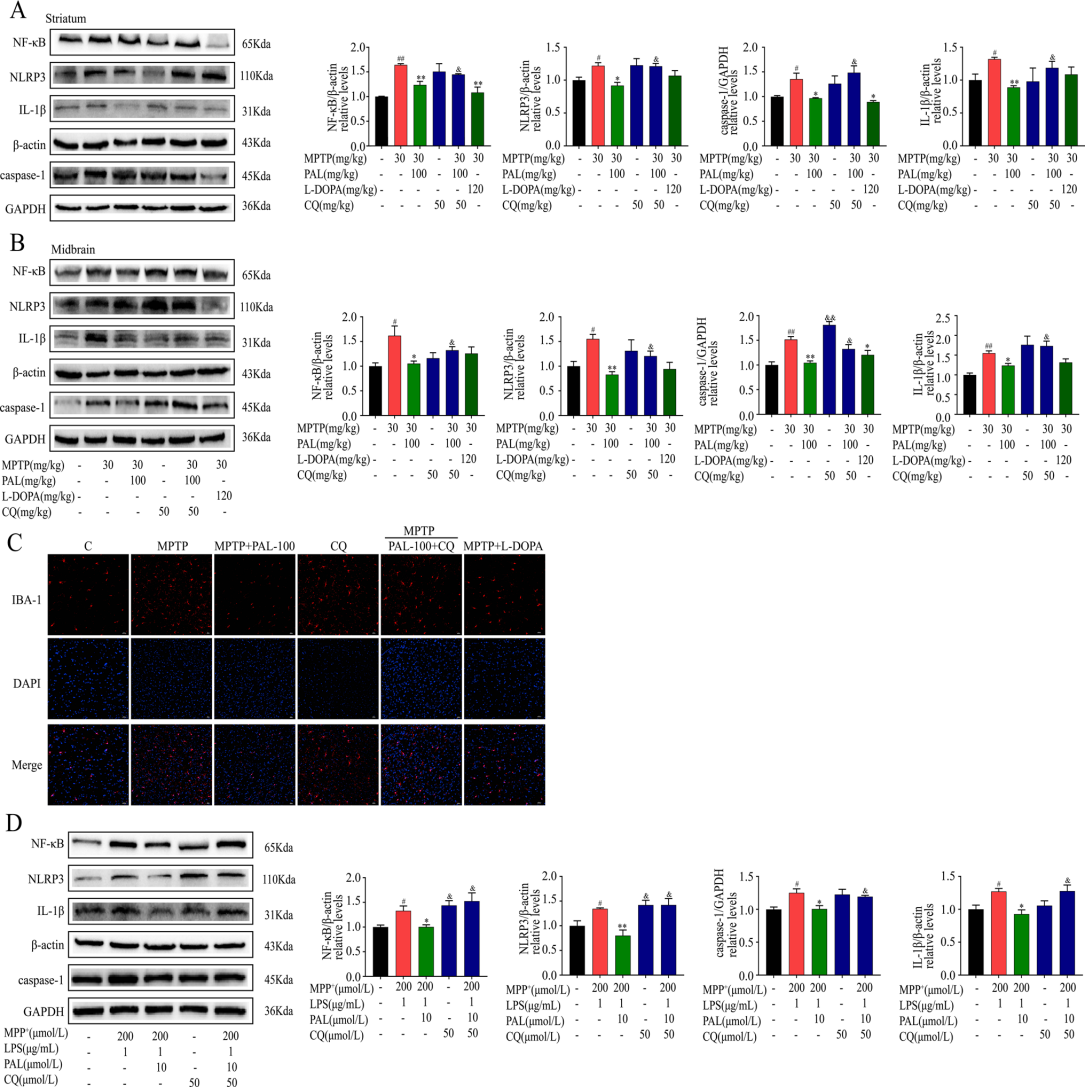
The inhibitory effect of PAL on NLRP3 inflammasome and microglia was abolished by CQ. (**A**) western blot was used to detect the expression of NF-κB, NLRP3, caspase-1 and IL-1β in the striatum. (**B**) western blot was used to detect the expression of NF-κB, NLRP3, caspase-1 and IL-1β in the midbrain. (**C**) Immunofluorescence detection of microglia. Scale bar, 20 μm. (**D**) The expression of NF-κB, NLRP3, caspase-1 and IL-1β in BV2 cells was detected by western blot. Quantified data are normalized to the control group (the control group value is equal to 1). Data are expressed as means ± SEM, (n = 3). ^#^ P < 0.05, ^##^ P < 0.01 vs the saline group. ^*^ P < 0.05, ^**^ P < 0.01 vs MPTP group, ^&^ P < 0.05, ^&&^ P < 0.01 vs MPTP + PAL-100 group

Mitophagy exhibits negative regulation of NLRP3 inflammasomes, inhibiting the inflammatory response. The above experimental data have demonstrated that PAL can inhibit both the NLRP3 inflammasome and microglia activation. Next, we further clarified the inhibitory effect of PAL on NLRP3 inflammasomes and microglia in the presence of CQ. Western blot results indicate that the inhibitory effect of PAL on the NLRP3 inflammasome is reduced, leading to a significant increase in the expression of NF-κB, NLRP3, caspase-1, and IL-1β, as well as microglia activation in the presence of CQ (Fig. 7A-C). Consistently, the addition of CQ to treat BV2 cells eliminated the inhibitory effect of PAL on the expression of NLRP3 and its associated proteins (Fig. 7D). Additionally, L-dopa administration does not significantly inhibit NLRP3 and its associated proteins (Fig. 7A-C).

## Discussion

PD is the fastest growing neurological disease, affecting approximately 6 million people worldwide [2, 46]. Existing studies suggest that it may be related to genetics, mitochondrial dysfunction, oxidative stress, and other factors; it is the result of the synergistic effect of multiple mechanisms. Among them, mitophagy [47, 48] and NLRP3 inflammasoma-mediated proinflammatory responses [49, 50] play an important role in the molecular pathogenesis of PD.

PAL, an isoquinoline alkaloid, has pharmacological effects, such as anti-inflammatory, neuroprotection [36, 51], and immunomodulatory effects. In LPS-induced gEECs, PAL treatment inhibited the release of tumor necrosis factor (TNF)-α and interleukin (IL)-1β. This inhibition was achieved by down-regulating the expression of Toll-like receptor 4 (TLR4), cluster of differentiation 14 (CD14), Toll/interleukin 1 receptor (TIR)-domain-containing adaptor protein inducing interferon-β (TICAM, TRIF), and nuclear factor-κB (NF-κB) [35]. In addition, PAL protected mice against DSS-induced colitis by inhibiting NLRP3 inflammasome activation through promoting autophagy [52]. Although previous studies have shown that PAL has various physiological effects, its role in PD has not been investigated. In the present study, we evaluated the effects of PAL on motor deficits and dopaminergic neuron loss in MPTP-induced PD mice and examined whether its effect is exerted by promoting mitophagy-mediated NLRP3 inflammasome inactivation.

1-methyl-4-phenyl-1,2,3,6-tetrahydropyridine (MPTP) induced PD mouse model is one of the widely used animal models for PD, which can partially reflect the pathogenesis and pathological features of PD [53, 54]. After intraperitoneal injection of MPTP for 7 consecutive days, mice showed marked motor dysfunction and a significant decrease in dopaminergic neurons, which is consistent with previous findings [55–57].

Mitophagy is a cellular process that clears damaged mitochondria, crucial for maintaining intracellular environmental stability and function [18]. In PD, mitochondrial damage is a key factor in disease progression. Existing research has found that impaired mitophagy leads to the accumulation of swollen and damaged mitochondria within cells, resulting in oxidative stress and the accumulation of cellular toxins, ultimately leading to the death of dopaminergic neurons [47]. Mutations in genes associated with PD, such as PINK1, Parkin, and LRRK2, impair mitophagy, further damaging dopaminergic neurons. Therefore, promoting mitophagy to clear damaged mitochondria, thereby reducing oxidative stress and cell death, is an important strategy for treating PD. Previous studies have shown that in PD, promoting PINK1-Parkin-mediated mitophagy can reduce the accumulation of damaged mitochondria, promote the colocalization of LC3 with mitochondria, and reduce the death of dopaminergic neurons [56, 58].

In this study, our results indicate that MPTP induced a significant decrease in the expression of LC3, P62, and PINK1 proteins. MPP^+^ induced a reduction in cell viability, a decrease in mitochondrial membrane potential, an increase in mitochondrial ROS, and a decline in the expression of mitochondrial autophagy-related proteins LC3, P62, and PINK1. This was accompanied by the activation of the NLRP3 inflammasome. Treatment with PAL significantly improved motor function deficits in PD mice, reduced the loss of dopaminergic neurons, reversed the decline in neuronal viability, restored mitochondrial membrane potential, suppressed mtROS generation, and promoted the expression of mitochondrial autophagy-related proteins. Additionally, PAL treatment inhibited the activation of the NLRP3 inflammasome.

Mitochondrial dysfunction and the failure of the electron transport system lead to the excessive production of mtROS and mtDNA, thereby enhancing the activation of the NLRP3 inflammasome. Excessive mtROS and the NLRP3 inflammasome, after the release of mtDNA into the cytoplasm, in turn promote LPS or ATP-induced release of IL-1β and IL-18 [59, 60]. Defects in mitochondrial fission and fusion induce mitochondrial damage, promote the colocalization of NLRP3 with mitochondria, and increase the activation of the NLRP3 inflammasome [61]. Targeting damaged or excess mitochondria through mitophagy to maintain a healthy intracellular mitochondrial state may be an effective means of regulating NLRP3 inflammasome activation. To further verify the inhibitory effect of PAL on the NLRP3 inflammasome through mitophagy, we treated mice with CQ. CQ significantly reversed the positive effects of PAL on the behavior of PD mice, eliminated the protective effects of PAL on dopaminergic neurons, and eliminated the inhibitory effects of PAL on the NLRP3 inflammasome and the activation of microglia.

Therefore, based on our data, we conclude that PAL ameliorates motor dysfunction and protects neurons from damage in MPTP mice. The accumulation of ROS in damaged mitochondria acts as an activation signal for the NLRP3 inflammasome. PAL inhibits the activation of the NLRP3 inflammasome by promoting mitophagy and prevents the death of dopaminergic neurons.

## Data Availability

No datasets were generated or analysed during the current study.
